# Multiscale Distribution Entropy and t-Distributed Stochastic Neighbor Embedding-Based Fault Diagnosis of Rolling Bearings

**DOI:** 10.3390/e20050360

**Published:** 2018-05-11

**Authors:** Deyu Tu, Jinde Zheng, Zhanwei Jiang, Haiyang Pan

**Affiliations:** School of Mechanical Engineering, Anhui University of Technology, Maanshan 243032, China

**Keywords:** multiscale distribution entropy, t-distributed stochastic neighbor embedding, Kriging-variable predictive models, rolling bearing, fault diagnosis

## Abstract

As a nonlinear dynamic method for complexity measurement of time series, multiscale entropy (MSE) has been successfully applied to fault diagnosis of rolling bearings. However, the MSE algorithm is sensitive to the predetermined parameters and depends heavily on the length of the time series and MSE may yield an inaccurate estimation of entropy or undefined entropy when the length of time series is too short. To improve the robustness of complexity measurement for short time series, a novel nonlinear parameter named multiscale distribution entropy (MDE) was proposed and employed to extract the nonlinear complexity features from vibration signals of rolling bearing in this paper. Combining with t-distributed stochastic neighbor embedding (t-SNE) for feature dimension reduction and Kriging-variable predictive models based class discrimination (KVPMCD) for automatic identification, a new intelligent fault diagnosis method for rolling bearings was proposed. Finally, the proposed approach was applied to analyze the experimental data of rolling bearings and the results indicated that the proposed method could distinguish the different fault categories of rolling bearings effectively.

## 1. Introduction

Rotating machinery is the core equipment of large petroleum and petrochemical industries and its faulty operation can lead to entire plant shutdowns and huge economic losses. Rolling bearings are an important part of rotating machinery and their working state is closely related to the operation reliability of rotating machinery. However, rolling bearings are also one of the most vulnerable parts of the machines. According to statistics, 70% of failures in rotating machinery are caused by the rolling bearings in the gearbox. Therefore, it is important and necessary to develop fault detection and diagnosis methods and technologies for rolling bearings.

The key of rolling bearing fault diagnosis is to extract the information related with fault location from the vibration signals. In recent years, the complexity of time series has served as an essential property for deeply understanding the non-stationary characteristics of vibration signals from mechanical systems [1,2]. Entropy-based complexity measurement methods such as approximate entropy (ApEn) [3,4,5], sample entropy (SampEn) [6,7,8], fuzzy entropy (FuzzyEn) [9,10] and permutation entropy (PE) [11,12] have been significantly important technologies to evaluate the dynamical complexity of time series. Generally, higher entropy indicates higher uncertainty and lower entropy indicates more regularity and certainty of a system [13,14]. The entropy-based methods have been widely applied to mechanical fault diagnosis and many research works of this topic have been published. For example, ApEn was successfully applied to extract the fault features from the vibration signals of mechanical equipment by Yan et al. [15]. SampEn was used to extract the fault features from signals of different kinds of bearings under different work conditions by Ding et al. [16] and the study indicated SampEn can effectively reflect the complexity of fault information changes. In [17] a fault diagnosis model for power transformers was put forward based on fuzzy entropy by Sheng et al. PE was applied to the early fault diagnosis for rolling bearings in [18] and the research indicated that early abnormal characteristics of vibration signals could be successfully detected by PE before any fault occurred. However, many recent studies have shown that the single scale entropy quantifies unessentially the randomness that may not be uniformly identical to complexity [19,20,21], i.e., single scale entropy is not effective to measure the complexity of time series [19].

To overcome the above limitation of sample entropy, multiscale entropy (MSE) was developed by Costa et al. [19] by using the coarse graining process at different scales. Zheng et al. [21] used MSE to analyze the mechanical vibration signals and found that MSE had a great advantage over single scale entropy. In [22] multiscale permutation entropy (MPE) was combined with SVM to the fault diagnosis of rolling bearingd by Zheng et al. and the research indicated that MPE can obtain a good diagnostic effect. In [23], Zheng et al. also proposed to use multiscale fuzzy entropy (MFE) to analyze the complexity of vibration signals of rolling bearings and they found that MFE can effectively extract the potential fault feature information. However, both the MSE and MFE algorithms are very sensitive to the similarity tolerance and data length. Distribution entropy (DistEn) proposed by Li et al. [24] takes full advantage of the inherent information underlying the vector-to-vector distances in the state space by probability density estimation and has relatively lower sensitivity to the predetermined parameters. Most of all, it shows much stability for quantifying the complexity of short time series.

Based on the above analysis and inspired by multiscale fuzzy entropy, in this paper a novel nonlinear dynamic method termed multiscale distribution entropy (MDE) is proposed for measuring the complexity of time series. Since vibration signals generated by machinery system are non-linear and non-stationary, MDE is able to measure the complexity of time-series in a multiscale temporal and spatial way through quantifying the amount of information contained in the inter-vector distances of the state space representation of time series. In this paper MDE is used to extract the fault information from the vibration signals of rolling bearings.

After extracting the features by using MDE, the obtained features are input to a multi-classifier for an intelligent fault diagnosis of rolling bearings. However, since the features extracted from vibration signals of rolling bearings are often high dimensional with information redundancy, which will cause a recognition rate decrease and a time-consuming training process, therefore, it is necessary to reduce the feature dimensions and select the most important sensitive features to construct low dimension features for reflecting the main fault information. The t-distributed stochastic neighbor embedding (t-SNE) manifold learning algorithm introduced by Laurens et al. [25,26] recently is a nonlinear manifold learning algorithm for deep learning problems. It is mainly based on the idea that if two data points are close in the original space while their mapping distance is far, they will attract each other. On the contrary, if two data points are far in the original space while their mapping distance is relatively close, they will reject each other. t-SNE is an impactful dimension reduction tool, which can reduce the feature dimensions and enhance the recognition rate overwhelmingly and in this paper it is utilized to reduce the feature dimension of rolling bearing vibration signals.

After extracting sensitive fault features by using t-SNE, naturally, a multi-fault classifier is needed to achieve the fault diagnosis of rolling bearing automatically and make a reliable decision quickly. In recent years, various pattern recognition methods have been applied to mechanical fault diagnosis, such as fuzzy C-means clustering, support vector machines (SVMs) [27,28], and extreme learning machine (ELM) [29,30], etc. Although the basic theories of these methods are successfully established, there are still some inherent deficiencies [31,32], i.e., they all almost ignore the intrinsic relationship among the features. Recently, variable predictive model based on class discrimination (VPMCD) was proposed by Raghuraj and Lakshminarayanan [33] for pattern recognition and prediction. VPMCD has been applied to mechanical fault diagnosis [34,35,36]. However, only four regression models are used for predicting in the original VPMCD and the prediction accuracy will be reduced when the relationships of feature values are more complex. Kriging function is an unbiased estimation model with minimum variance and is usually composed of three regression models and seven relevant models. Then KVPMCD is proposed by replacing the regression model of VPMCD with a Kriging function. KVPMCD method has overcome the monotonicity of VPMCD model and the Kriging function-based model is superior to the existing models of VPMCD and has a stronger adaptability than the original VPMCD. Hence, KVPMCD can be used to reflect the complex relationship among feature values [37,38]. Then based on MDE, t-SNE and KVPMCD, a novel fault diagnosis approach for rolling bearings was proposed in this paper.

The rest of this paper is organized as follows: in Section 2 the definition of DistEn and its parameter selections are introduced and then MDE algorithm is proposed. In Section 3 MDE is compared with MSE by analyzing simulation signals. In Section 4 a new fault diagnosis method of rolling bearings is proposed based on the MDE, t-SNE and KVPMCD and is applied to analyse rolling bearing experimental data. Conclusions are drawn in Section 5.

## 2. Algorithms of DE and MDE

### 2.1. Definition of DistEn

DistEn was defined to measure the complexity of univariate signal by quantifying a lot of state information [24], which is covered in inter-vector distances of state space representation of signal.

For a given time-series {*u*(*i*)} = {*u*(1), *u*(2), *u*(3), …, *u*(*N*)}, the detailed DistEn algorithm can be introduced as follows:

• *State space reconstruction*

(N−(m−1)δ) vectors X(i) can be constructed by *X*(*i*) = {*u*(*i*), *u*(*i* + *δ*), …, *u*(*i* + *m* − 1) × *δ*}, 1≤i≤N−(m−1)δ. Here *m* and δ represent the embedding dimension and time delay, respectively. There are several methods for selecting the time delay for a univariate signal, such as mutual information and auto-correlation [39,40].

• *Distance matrix construction*

The inter vector distances (any feasible combinations of X(i) and *X*(*j*) by *d_i,j_* = max(|*u*(*i* + *k*)− *u*(*j* + *k*)|, 0 ≤ *k* ≤ *m* − 1) are computed for all 1≤i,j≤N−m. The distance matrix is defined as *D* = {*d_i,j_*}.

• *Probability density estimation*

The empirical probability density function of the distance matrix *D* is estimated by the histogram way to a determine number *M*, where *p_t_*(*t* = 1, 2,…, *M*) is frequency and the estimated value are not included in the distance matrix *D* while *i* = *j*.

• *DistEn Calculation*

The *DistEn* of a given signal {u(i)} can be calculated by:

(1) $$DistEn(m,\delta ,M)=-\frac{1}{\log_{2}(M)}\sum_{t=1}^{M}p_{t}\log_{2}(p_{t})$$

### 2.2. DistEn Parameter Selection 

The calculation of *DistEn* is related with *m* and *M*. Actually, *M* represents an intermediate parameter and is like the similarity tolerance *r* in SampEn. *M* is recommended to set a value between 512 and 1024 [24]. *r* could be selected between [0.1*SD*, 0.25*SD*] (*SD* stands for standard deviation) [41], respectively. In this paper, we set m=2 and M=512 in the calculation of *DistEn*.

### 2.3. Multiscale Distribution Entropy

The proposed MDE approach incorporates two steps: (1) the given time series is divided into multiscale time series by conducting a moving-averaging procedure [42]; (2) the complexity of the moving-averaged time series over scale factor *τ* are quantified by using *DistEn*.

1. For a given discrete time series $x={\left\{x_{i}\right\}}_{i=1}^{N}$, let $z_{j}^{\tau }$ represents the moving-averaged time series at a scale factor *τ* that is constructed by:

(2) $$z_{j}^{\tau }=\frac{1}{\tau }\sum_{i=j}^{j+\tau -1}x_{i},1\leq j\leq N-\tau +1$$

The length of moving-averaged time series over *τ* is $\tilde{n}(\tau )=N-\tau +1$.

2. The *DistEn* is calculated of the moving-averaged time series $z^{\tau }$ by:

(3) $$\mathrm{MDE}(x,m,\tau ,M)=DistEn(z^{\tau },m,\delta =\tau ,M)$$

As a complexity measurement method, similar to MFE [43,44], MDE measures the complexity of time-series by quantifying rich information contained in time series over multiple scales. Besides, compared with the traditional methods, MDE is insensitive to the input parameters especially the length of the time series, which will be verified by the following simulation experiments.

## 3. Comparison Analysis of MSE and MDE

### Simulation Tests

**Figure 1:**
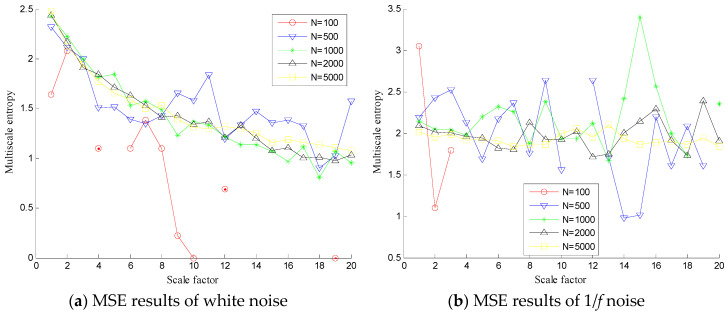
MSE results of white noise (**a**) and 1/*f* noise (**b**) with different data lengths.

**Figure 2:**
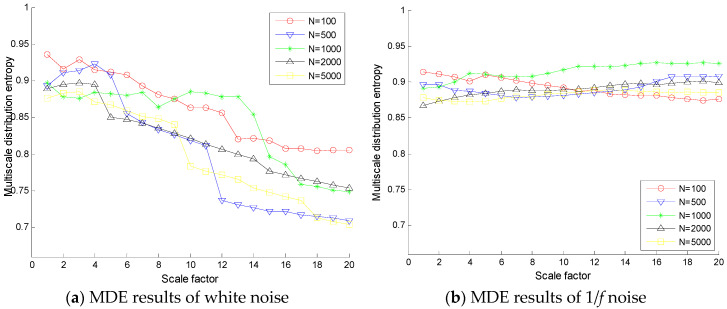
MDE results of white noise (**a**) and 1/*f* noise (**b**) with different data lengths.

In order to investigate the effect of data length on MSE and MDE, we firstly test MSE by analyzing simulated white noises and 1/*f* noises with different data lengths (*N* = 100, 500, 1000, 2000 and 5000) and the results are given in Figure 1a,b. As shown in Figure 1a,b, with the increase of scale factor, the MSE of white noise will decrease and have large fluctuations in the larger scale factor while the MSE of 1/*f* noise fluctuates near a fixed value. Besides, for a short time series with length less than 2000 points, the MSE in some scales are undefined (especially for the larger scale factors). Figure 2a,b show the MDE curves of white noise and 1/*f* noise, respectively. It can be seen clearly from Figure 2a,b that with the increase of the scale factor, the MDE curve of white noise decreases gradually while that of 1/*f* noise is stable standing at a constant level. Also the entropy of MDE in every scale is defined. Therefore, MDE shows much better precision than MSE when analyzing shorter time series.

**Figure 3:**
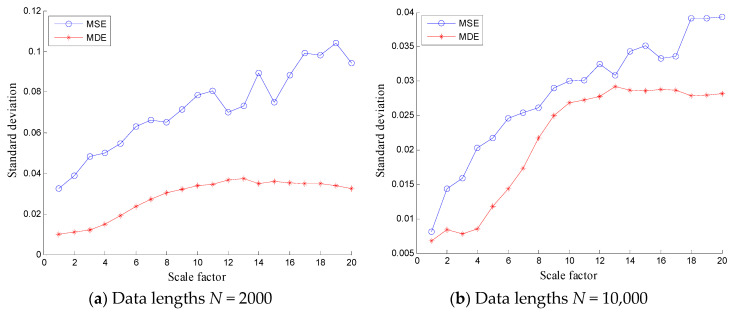
Comparison of standard deviations between MSE and MDE of white noise with data lengths (**a**) *N* = 2000 and (**b**) *N* = 10,000.

**Figure 4:**
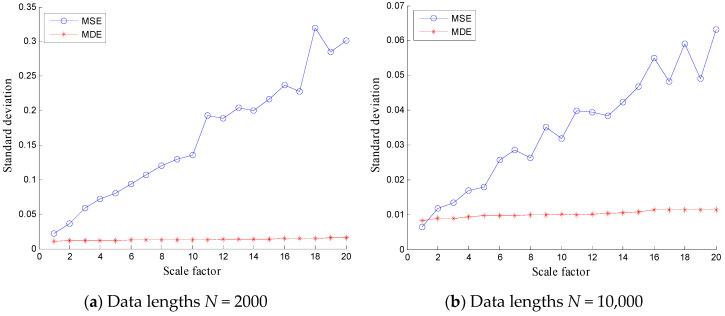
Comparison of standard deviations between MSE and MDE of 1/*f* noise with data lengths (**a**) *N* = 2000 and (**b**) *N* = 10,000.

To verify the stability of the proposed method, MSE and MDE of white noises and 1/*f* noises are calculated and the results are shown in Figure 3 and Figure 4 with different lengths (*N* = 2000 and 10,000), where 100 independent noise signals are calculated. It can be found from Figure 3 and Figure 4 that the SDs of MSE of white noises and 1/*f* noises are relatively larger than that of MDE over all scale factors. Hence, the SDs of MDE are more consistent and accurate than those of MSE for white noises and 1/*f* noises with different lengths.

The above analysis results indicate that the MSE estimation is sensitive to data length and its SD values manifest an increasing trend with the data length decreasing while the computation of MDE is independent on data length. Therefore, MDE algorithm is more precise and consistent than MSE.

## 4. The Proposed Fault Diagnosis Approach and Its Applications

### 4.1. t-SNE Algorithm

When we have extracted MDEs from vibration signals of rolling bearing, it is necessary to select the most important sensitive features to construct the sensitive fault features for intelligent fault diagnosis. In this paper t-SNE is utilized to reduce feature dimensions and its mainly steps are described as follows:

(1) For an original data sequence $X=\{x_{1},x_{2},\ldots ,x_{n}\}$, the joint probabilities $p_{ij}$ are defined to measure the pairwise similarity between objects $x_{i}$ and $x_{j}$. The pairwise affinities $P_{j}|i$ with perplexity (*Perp*) are calculated according to Equation (4). The perplexity *Perp* is as a cost function parameter:

(4) $$P_{j}|i=\frac{\exp\left(-\frac{\|x_{i}-x_{j}{\|}^{2}}{2\sigma_{i}^{2}}\right)}{\sum_{k\neq i}\exp\left(-\frac{\|x_{i}-x_{k}{\|}^{2}}{2\sigma_{i}^{2}}\right)}$$

where $\sigma_{i}$ is Gauss variance of data point $x_{i}$.

(2) Once the data point $x_{i}$ is an outlier, it will cause that the position of map point to not be well determined by the positions of the other map points. To deal with this issue, the joint probabilities $p_{ij}$ are defined in the high-dimensional space as symmetry conditional probabilities, so we set $p_{ij}=\frac{p_{j}|i+p_{i}|j}{2n}$, where *n* is the total number of data points. 

(3) Let the mapping points of the high-dimensional space data points $x_{i}$ and $x_{j}$ in the low-dimensional space are $y_{i}$ and $y_{j}$. In order to satisfy $p_{ij}$ = $q_{ij}$, where $q_{ij}$ is the joint probabilities in low dimensional space, then the distance in the low-dimensional space should be slightly smaller for the points closer in the high-dimensional space. Also the distance in the low-dimensional space should be farther for the points that are far apart in the high-dimensional space. Hence, the joint probabilities $q_{ij}$ in low dimensional space are defined as Equation (5) by using a Student t-distribution with one degree of freedom in t-SNE.

(5) $$q_{ij}=\frac{{\left(1+\|y_{i}-y_{j}{\|}^{2}\right)}^{-1}}{{\sum_{k\neq l}\left(1+\|y_{k}-y_{l}{\|}^{2}\right)}^{-1}}$$

(4) To measure the similarity between high-dimensional space joint conditional probability distribution *P* and low-dimensional space joint conditional probability distribution *Q*, and by gradient descent algorithm minimizing cost function $C=\sum_{i}KL(P_{i}\|Q_{i})=\sum_{i}\sum_{j}p_{j|i}\log\frac{p_{j|i}}{q_{j|i}}$ that Kullback–Leibler divergence between *P* and *Q*, the gradient $\delta C/\delta y_{i}$ is calculated according to Equation (6):

(6) $$\frac{\delta C}{\delta y_{i}}=4{\sum_{j}\left(p_{ij}-q_{ij}\right)\left(y_{i}-y_{j}\right)\left(1+\|y_{i}-y_{j}{\|}^{2}\right)}^{-1}$$

(5) Low dimensional data can be obtained according to Equation (7)

(7) $$y^{\left(t\right)}=y^{\left(t-1\right)}+\eta \frac{\delta C}{\delta y}+\alpha (t)\left(y^{\left(t-1\right)}-y^{\left(t-2\right)}\right)$$

where learning rate η and momentum α(t) are optimization parameters.

(6) Iterate loop stepts (3) to (5) until *t* from 1 to *T*, where *T* is maximum number of iterations that should be pre-set. Finally low dimensional data *y*^(*T*)^ = {*y*_1_, *y*_2_,…, *y_n_*} are obtained.

The cost function in t-SNE algorithm is different from SNE in two aspects: (1) t-SNE using symmetric SNE cost function of reduced gradient; (2) It uses the t-student distribution instead of the Gaussian distribution to calculate the similarity between two points in a low dimensional space. The heavy tailed distribution in low dimensional space is employed in t-SNE to slow down the aggregation and optimization of SNE. t-SNE as a nonlinear dimensionality reduction algorithm for deep learning, the structure of low dimensional manifold can be recovered from high-dimensional data, hence it can achieve dimensionality reduction and data visualization.

### 4.2. KVPMCD

#### 4.2.1. Basic Concepts and Frameworks of KVPMCD

The Kriging model assumes that the real relationship of the response value of the system and independent variable can be expressed as follows:

(8) f(x)=g(x)+z(x)

where *g*(*x*) represents the deterministic drift and *z*(*x*) represents fluctuation. z(x) provides an approximation to the simulated local bias and is a function associated with the relevant model.

The regression models have three kinds of forms in the Kriging model, i.e., zero order polynomial, one order polynomial and two order polynomial. They are the main framework of the established Kriging model. The essence of VPMCD is to make use of the intrinsic relationship among features, and the approximate model is established by calling the existing four regression models to reflect the real relationship between eigenvalues. However, when the relationship among eigenvalues is complex, the models in the VPMCD may not fully reflect the internal connection among the eigenvalues. However, there are three kinds of regression models and seven kinds of relevant models in KVPMCD, i.e., exponential model, generalized exponential model, Gaussian model, linear model, spherical model, cubic model and spline model. In Kriging model, they can be combined to build 21 kinds of models to make the established model more realistic. Therefore, KVPMCD is obtained by applying the Kriging model to VPMCD.

#### 4.2.2. Kriging Model-Based KVPMCD Method

The main steps of KVPMCD can be described as follows:

(1) For *g* class classification problem, *n* training samples are collected and each sample number is $n_{1},n_{2},\ldots ,n_{g}$. The feature *X* = [$x_{1},x_{2},\ldots ,x_{p}$] is extracted from all training samples and the size of each feature is $n_{1}\times p,n_{2}\times p,\ldots ,n_{g}\times p$ respectively.
(2) The feature $X_{i}$ (*i* = 1, 2, …, *p*) of the *k*th (1 ≤ *k* ≤ *g*) training sample is selected as the predicted variable and the remaining *p*-1 feature $X_{j}$ (*j* ≠ *i*) is seen as predictive variables.
(3) Let the regression model type *z* = 1 (1 ≤ *z* ≤ *Z*) (zero, one and two order polynomial. Three models are marked as 1, 2, and 3, respectively). The model category of the relevant models is *h* = 1 (1 ≤ *h* ≤ *H*) (exponential, generalized exponential, Gaussian, linear, spherical, cubic, spline, respectively, is marked as 1, 2, 3, 4, 5, 6 and 7), and then a mathematical model is established.
(4) Set *h* = *h* + 1 and *z* = *z* + 1, respectively, until *h* = *H*, *z* = *Z*. The combination of predictive variables is common to *H* × *Z* species. Therefore, $n_{k}$ = *H* × *Z* mathematical equations can be established.
(5) $n_{k}$ equations can be established for each feature set $X_{i}$. The feature of each training sample in the *k*th class can be obtained. The predicted value $X_{ipred}$ of the feature $X_{i}$ can be obtained by the Kriging model.
(6) To calculate the prediction error square sum $SSE_{l}=\sum_{v=1}^{n_{k}}{\left(X_{iv}-X_{ivpred}\right)}^{2}$ of the $n_{k}$ variable prediction model respectively, where *v* represents the *v*th training sample and $l=1,2,\ldots ,n_{k}$. The variable prediction model corresponding to the minimum value of $SSE_{l}$ is selected as the variable prediction model $VPM_{i}^{k}(i=1,2,\ldots ,p)$ of the feature $X_{i}(i=1,2,\ldots ,p)$ in the *k*th class training sample. Then save the corresponding model parameters and predictive variables.
(7) Let *k* = *k* + 1, repeat steps (3)~(6) until *k* = *g*. At this point, in the case the variable prediction model $VPM_{i}^{k}$ are established for all the feature of g categories respectively, where *k* (*k* = 1, 2, …, *g*) denotes category label and *i* (*i* = 1, 2, …, *p*) represents the feature. These variable predictive models form a VPM matrix with size of *g* × *p*.
(8) All training samples are constructed as a test sample set to perform a return classification test for each VPM matrix. The regression model type and the relevant model type corresponding to the VPM matrix with the highest correct classification rate are selected as the type of the best variable prediction model.
(9) The feature X = [$x_{1},x_{2},\ldots ,x_{p}$] are extracted for the selected test sample set. For all the feature values $X_{i}(i=1,2,\ldots ,p)$ of the test sample, respectively, the variable prediction model $VPM_{i}^{k}$ is employed to predict it, and the predicted value $VPM_{ipred}^{k}$ is obtained.
(10) The squared sum $SSE^{k}=\sum_{i=1}^{p}{\left(X_{i}-X_{ipred}^{k}\right)}^{2}$ of the predicted errors is calculated for all features in the same category. And the minimum $SSE^{k}$ is used as the discriminant function to classify the test samples.

As an effective automatic pattern classifier, KVPMCD is different from traditional classifiers that ignore the intrinsic links between variables. It establishes more realistic models, which reflect relationship between the variables and improve the prediction accuracy of VPMCD [37].

### 4.3. The Proposed Fault Diagnosis Method

Based on the advantages of MDE, t-SNE and KVPMCD, a novel fault diagnosis method of rolling bearing can be summarized as follows:

(1) Assume that the states of rolling bearing contain *K* classes and each state is collected by *N* groups. MDE of each vibration signal is computed with parameters *m* = 2, *δ* = 1, *M* = 512 and the maximum scale factor $\tau_{\max}$. $\tau_{\max}$ eigenvalues were obtained to represent the fault information of the vibration signals of rolling bearing in each group and the feature vector matrix $R^{N\times \tau_{\max}}$ is constituted, which can adequately digs out the characteristics information of different complex time series.
(2) t-SNE is used to reduce the dimension of feature vector matrix and a low dimensional sensitive feature set *R^N×i^* can be obtained, where *N* represents the number of samples and *i* represent the dimensions after dimensionality reduction.
(3) Training samples are composed of the selected 1/2 *N* group of each state randomly, the rest as test samples. The training samples are input to the KVPMCD based multi-classifier for training. The predictive model ${KVPM}_{i}^{k}$. is established, where *k* (*k* = 1, 2, …, *g*) represents different categories, *i* (*i* = 1, 2, …, *p*) represents different characteristic values.
(4) The outputs of classifier are used to diagnose the fault types of rolling bearing.

**Figure 5:**
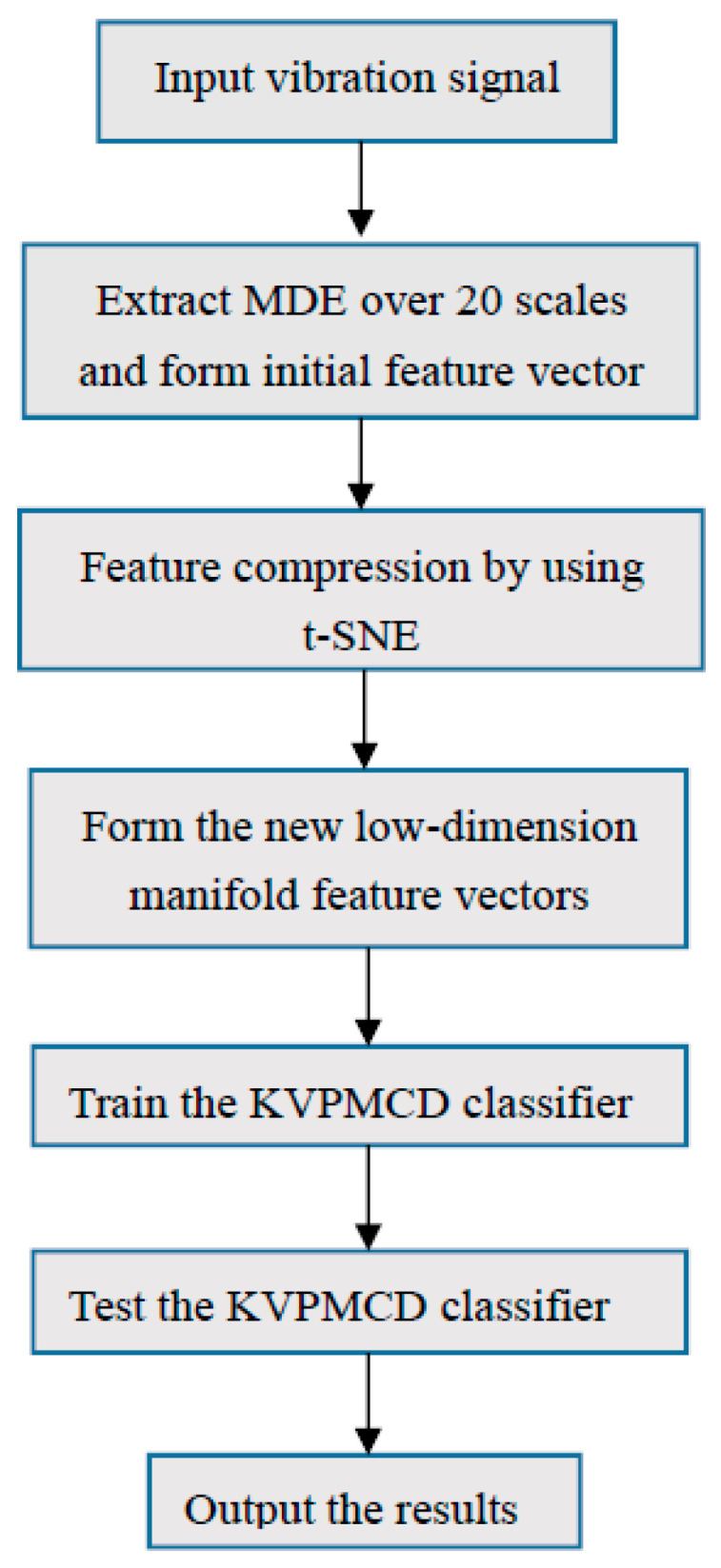
Flowchart of the proposed method.

The flowchart of proposed method is given in Figure 5.

### 4.4. Experimental Data Analysis

**Figure 6:**
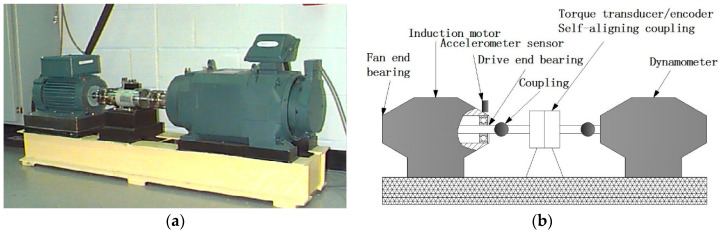
(**a**) The rolling bearing experiment system [45] and (**b**) its sketch.

In this part, the rolling bearing data kindly provided by the Case Western Reserve University (CWRU) Bearing Data databas are employed to test the effectiveness of MDE and the proposed approach. The collection apparatus and description of the bearing data are given in [45] and the experimental system is depicted in Figure 6. The 6205-2RS JEM deep groove ball bearings (SKF, Göteborg, Sweden) were employed in the experimental test and electro-discharge machining was used to obtain test bearings with single point faults. A 2-horsepower motor and a dynamometer are contained in the data collection system and they were connected by a torque transducer. In order to collect the bearing vibration signals, an accelerometer with a bandwidth up to 5000 Hz was fixed on the motor housing at the drive end of the motor. The data collection system with a high-bandwidth amplifier lay out for rolling bearing vibration signals, and the sampling frequency of a data recorder up to 12,000 Hz per channel and the motor speed is 1730 r/min with loading 2.2 KW. The vibration signals of rolling bearing with four conditions were collected, including the normal condition, the ball fault condition (BF), the outer race fault condition (ORF) and inner race fault condition (IRF) [45].

**Figure 7:**
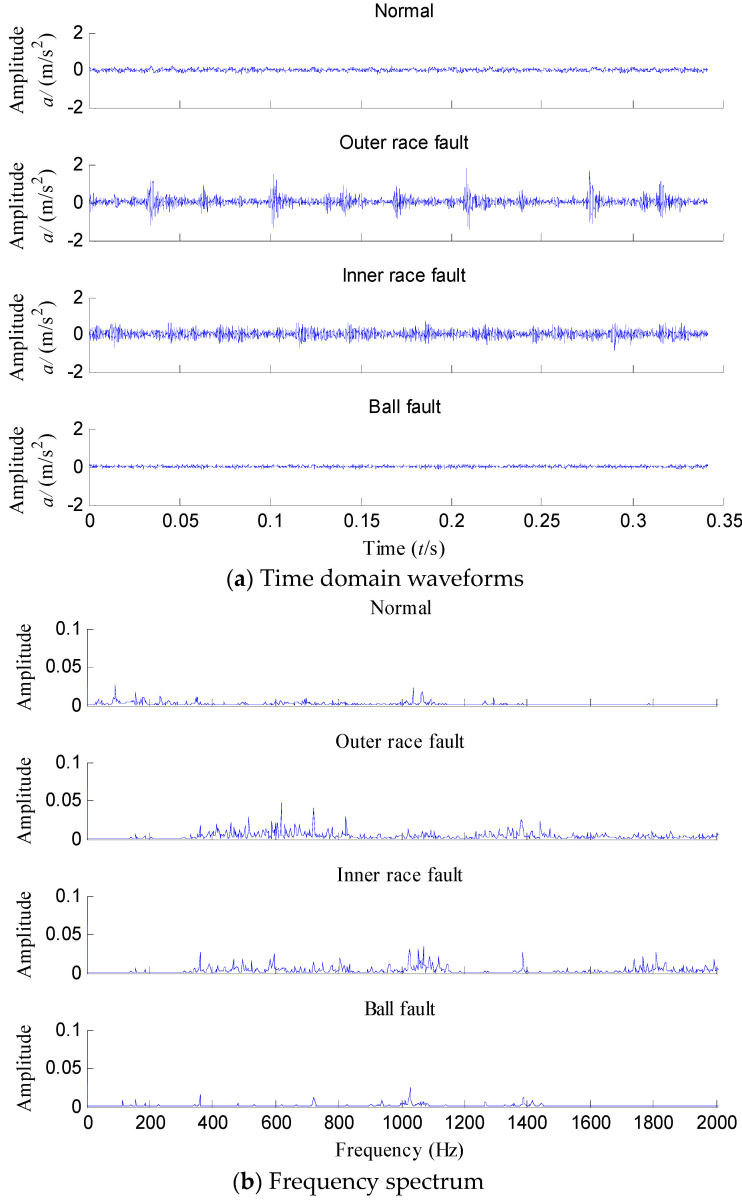
(**a**) Time domain waveforms and (**b**) frequency spectrum of rolling bearing vibration signal under four different conditions.

In the following part, 29 samples of each condition are used and the data length of each group sample is 4096. Fifteen samples of each category are randomly selected as the training data and the remaining 14 ones are used for testing. The time domain waveforms of vibration signals and their frequency spectrum under four fault categories are depicted in Figure 7a,b, respectively. It is difficult to distinguish the fault categories from their time domain waveforms and their frequency spectrum and it is unreliable to make decision according to the time domain waveforms and frequency spectrum.

**Figure 8:**
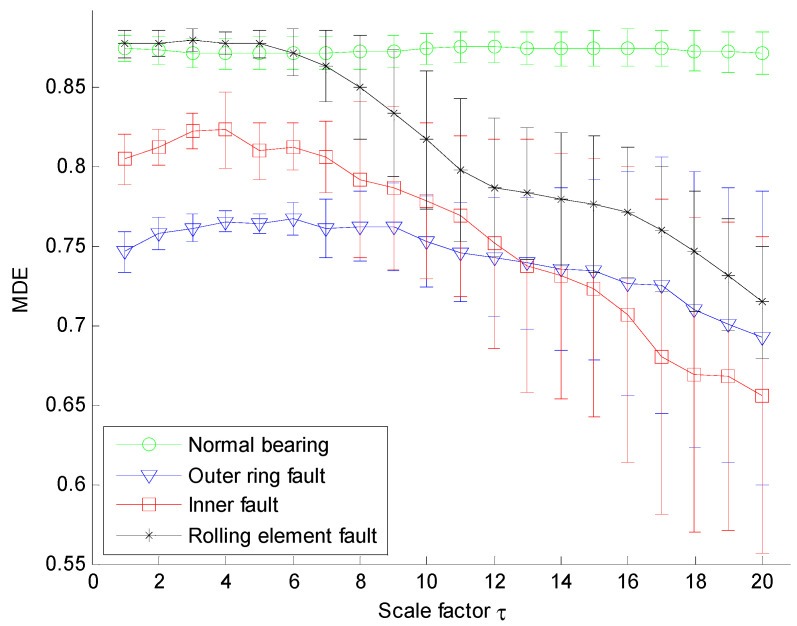
MDE over 20 scales of signals shown in Figure 7.

Next, the proposed MDE method is employed to analyze the vibration signals and the corresponding MDE curves are shown in Figure 8, where the error bars indicate the standard deviation. As can be seen from Figure 8 the four conditions of rolling bearings can be significantly distinguished by their MDE curves. The following conclusions can be reached: firstly, the MDE of vibration signals collected from normal bearings is constant at a fixed value with the increase of scale factor and is larger than that of vibration signals collected from faulty bearings over most scales. The MDE curves of vibration signals from faulty rolling bearings are all gradually decreasing, which indicates that the vibration signals of normal bearings are more complex compared with the signals collected from defective rolling bearings. This can be explained by the fact that when the rolling bearing works under healthy conditions, the vibration is random similar to the 1/*f* noise and thus has much higher complexity over multiple coarse grain time series. Once the rolling bearings work with local faults, the fault location will be a fixed exciting source of the system, which results in the fact that the similarity of their vibration signals increases and correspondingly, the complexity of vibration signals will decrease gradually. Therefore, MDE is an effective method to distinguish the healthy rolling bearings from faulty ones.

**Figure 9:**
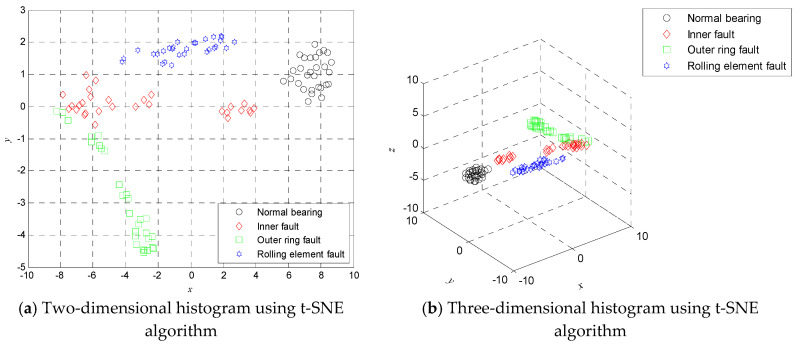
Two-dimensional histogram (**a**) and three-dimensional histogram (**b**) using t-SNE algorithm.

Nevertheless, if we take all MDE values over 20 scales as the features for training and testing, which will cause time consuming and computational complexity and even to some extent decrease the classification accuracy. Due to the fact the effective feature information is masked by high dimensional data, the t-SNE is employed to compress the high dimension data into low dimensional mapping to dig out the low dimensional manifold feature. Figure 9 shows the two-dimensional manifold diagrams and three-dimensional manifold diagrams obtained by using the t-SNE algorithm, where *x* denotes the first dimension coordinate, *y* denotes the second dimension coordinate, and *z* denotes the third dimension coordinate. Figure 9 shows that the features of each category can be separated in two-dimensional or three-dimensional space.

**Figure 10:**
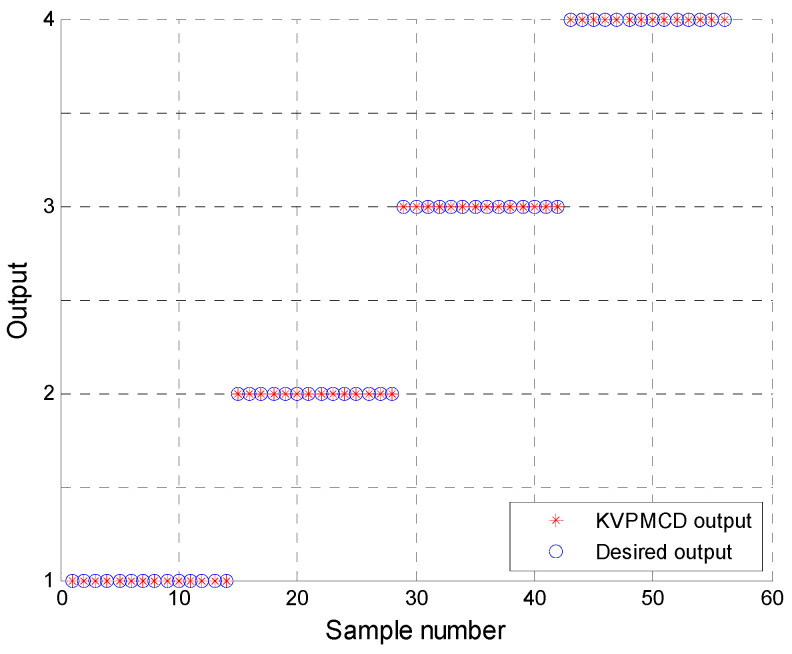
KVPMCD outputs of the proposed method.

Naturally, the low dimensional features are obtained as new sensitive fault features and 60 randomly selected samples are taken as the training data from the low dimensional data set and the remaining 56 samples are used as the testing data. Then the training data are used to train the KVPMCD classifier. After that the final ${KVPM}_{3}^{4}$. are obtained. Then the testing data set is used to validate the accuracy of ${KVPM}_{3}^{4}$. models for fault diagnosis. The results of the proposed method for the testing data of KVPMCD classifier are shown in Figure 10, where it can be seen that all the training and testing data are classified into the right category and the total recognition rate of the proposed approach is 100%, which indicates that the proposed approach provides a good classification result and is effective in rolling bearing fault diagnosis.

**Figure 11:**
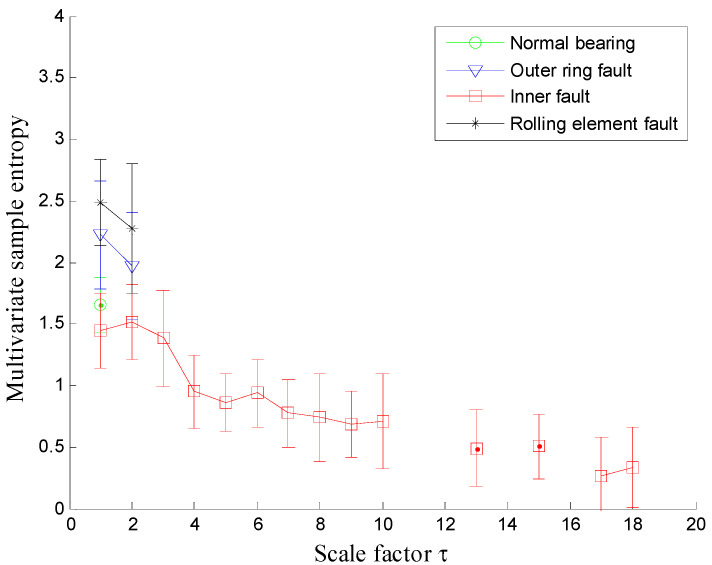
MSE of vibration signals with length 150 points.

**Figure 12:**
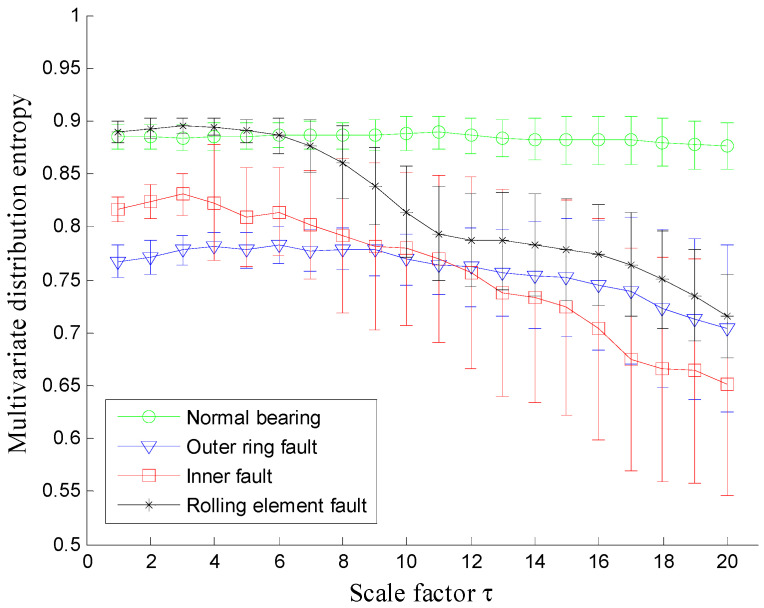
MDE of vibration signals with length 150 points.

To verify the advantage of MDE in analyzing shorter time series, MSE and MDE of each sample with 150 data points of four states of rolling bearing are extracted and the results are shown in Figure 11 and Figure 12. As can be seen from Figure 11, the MSE values on most scales are undefined. Hence, when the time series length is too short, MSE will lose its function in analyzing vibration signals of rolling bearings. The computation of MSE is heavily dependent on the length of data. It can be seen from Figure 12 that MDE are defined on all scales and the results are very similar to the results of Figure 8, where the length of the used data set is 4096, which indicates that unlike MSE, MDE is not sensitive to the length of data. In addition, MSE takes 8.85 s to analyze the same sample with 150 data points, which is longer than required by MDE (7.52 s), when the laptop memory is 4 GB, the processor is an Intel(R) Core(TM) i5-4200U CPU @ 1.60GHz and MATLAB version R2017a used).

**Figure 13:**
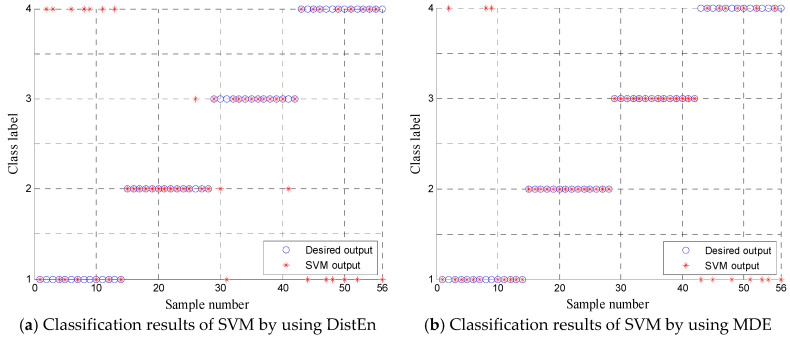
Comparison of the classification results of SVM between the DistEn (**a**) and (**b**) MDE.

To validate the superiority of multiscale analysis, the features obtained by using DistEn, i.e., the MDE with scale factor τ=1 is input to SVM classifier for discriminating the four kinds of rolling bearing fault and the output results of the testing samples are shown in Figure 13a. As we can see from Figure 13a that 17 testing samples are misclassified and the recognition rate is only 69.6429%. Also, the feature consisting of MDE in single scale factor τ=8 also is input to SVM classifier for training and testing and the output results are shown in Figure 13b with fault diagnosis rate 82.1429%. The comparison results reveal that the single scale analysis cannot discriminate the four kinds of rolling bearing working status and it cannot reflect the potential dynamic characteristics of the rotating machinery system. Therefore, it is necessary to carry out multiscale analysis by using MDE.

**Figure 14:**
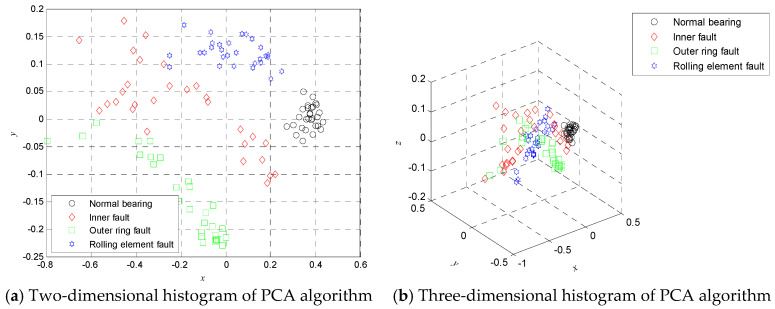
Two-dimensional histogram (**a**) and three-dimensional histogram (**b**) using PCA algorithm.

**Figure 15:**
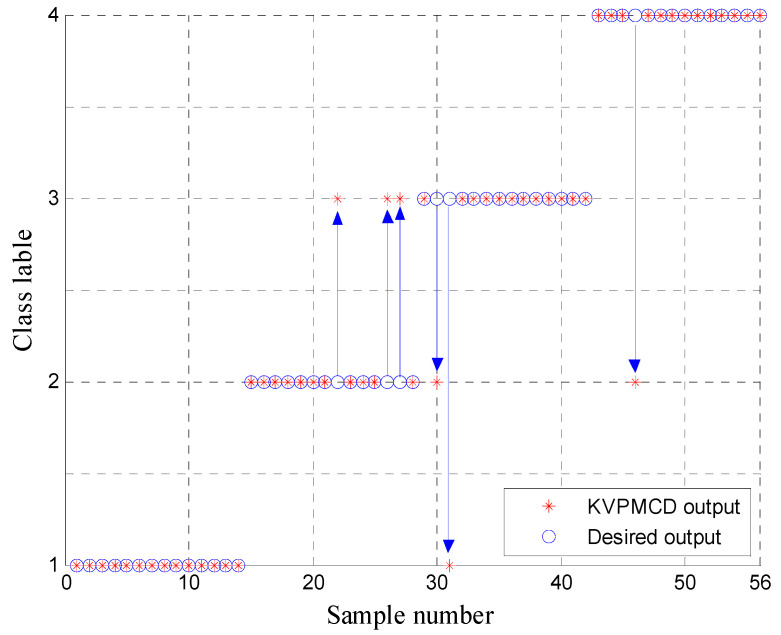
Outputs of KVPMCD classifier with features consisting of DistEns in scales: 1st, 8th and 15th.

**Figure 16:**
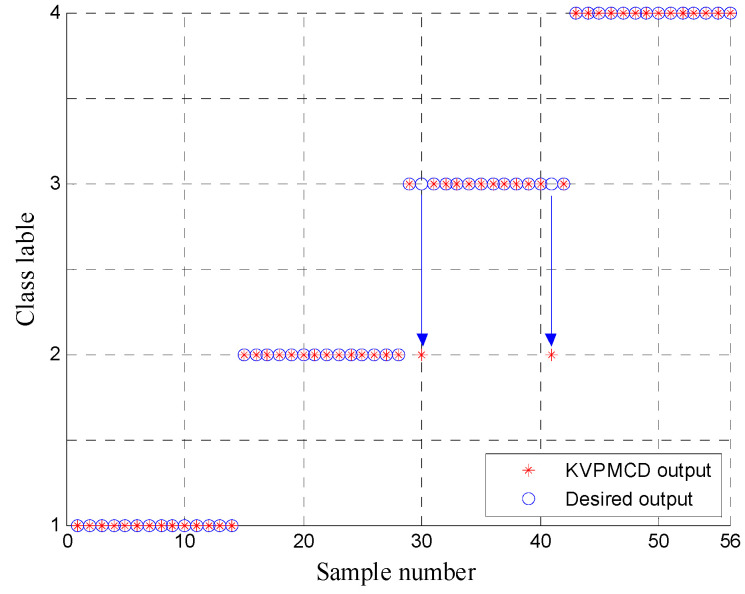
Classification results of KVPMCD by using PCA algorithm.

To verify the necessity and superiority of t-SNE for dimension reduction, firstly, the MDE in 20 scale factors are extracted from all samples and the MDE of training and testing data are input to KVPMCD for training and testing, respectively. The corresponding recognition rate is 94.6429%, which is lower than that of the proposed t-SNE based method. Secondly, in order to compare with the features random selection and the linear dimensionality reduction method, the DistEns in scales 1, 8, 15 are randomly selected to form the features and low dimensional features are obtained by using principal component analysis (PCA) showed in Figure 14, and then features obtained from the above two methods are input to the KVPMCD classifier for training and testing, respectively. Finally, the KVPMCD outputs of the testing data are given in Figure 15 after taking the random selection way in the same process of the above under same condition. It is obviously seen from Figure 15 that three testing samples with outer ring faults are misclassified as inner race faults and in two testing samples with inner race faults one is misclassified as an outer ring fault, while the other is misclassified as a normal bearing and one testing sample with a rolling element fault is misclassified as an outer ring fault. The overall recognition rate was 89.2857%, which is lower than that of the proposed method. The KVPMCD outputs of the testing data are presented in Figure 16 by using the PCA algorithm. It can be clearly observed from Figure 16 that we have two test samples with inner race faults that are misclassified as outer ring faults. The overall recognition rate reaches 96.4286%, but is also lower than that of the proposed method. Hence, the above analysis result indicates that it is necessary to carry out dimensionality reduction and t-SNE is integrant and overwhelmingly superior to the PCA algorithm.

Finally, to verify the superiority of KVPMCD, the VPMCD and SVM are used to construct a multi-fault classifier to deal with the above four-class classification problem. The obtained features are compressed to three-dimensions by using the t-SNE manifold learning algorithm and all samples are divided into training and testing ones. The recognition rates of the VPMCD- and SVM-based classifiers are 98.21% and 87.50%, respectively, which indicates that compared with VPMCD and SVM, the KVPMCD-based classifier gets the highest recognition rate and the result indicates the advantages of KVPMCD. The recognition rates of all used methods are summarized in Table 1, where τ represents the scale factor.

## 5. Conclusions

A novel nonlinear dynamic method termed MDE is proposed for measuring the complexity of time series. MDE is compared with the often used MSE method by analyzing artificial signals and the results indicates that MDE shows much better precision than MSE for the analysis of shorter time series. Based on MDE, t-SNE for feature selection and KVPMCD, a new fault diagnosis approach for rolling bearings is proposed. The proposed method is also applied to experimental data of rolling bearings for comparison with the existing MSE-based methods. The MDE is compared with single scale DE and the result shows the necessity of multiscale analysis. Besides, the t-SNE manifold learning algorithm for feature dimension reduction is contrasted with the most common used PCA and random selection methods and the results verify the advantages of the t-SNE algorithm in dimension reduction. Lastly, the recently proposed classification method KVPMCD is introduced to achieve rolling bearing fault diagnosis automatically and also compared with SVM and the original VPMCD. The comparison results show the superior fault identification rate of KVPMCD.

## Figures and Tables

**Table 1:** Recognition rates of different methods.

Methods	Accuracy Rate (%)
DistEn + SVM	69.64
MDE (the first 8 scales) + SVM	82.14
MDE (all 20 scales) + KVPMCD	94.64
MDE (three DEs in 1, 8 and 15 scales) + KVPMCD	89.29
MDE + PCA + KVPMCD	96.43
MDE + t-SNE + KVPMCD	100
MDE + t-SNE + VPMCD	98.21
MDE + t-SNE + SVM	87.50
